# Insights into milk microbiota differences among healthy, mastitis-suspected, and subclinical mastitis cows at dairy farms in Southern Vietnam

**DOI:** 10.5455/javar.2026.m1024

**Published:** 2026-03-16

**Authors:** Thuong Thi Nguyen, Cuong Kien Nguyen, Phong Dinh Tran, Khang Nguyen Duong, Hai Thanh Nguyen

**Affiliations:** 1 Faculty of Animal Science and Veterinary Medicine, Nong Lam University, Ho Chi Minh City 700000, Vietnam; 2 Graduate School of Environmental and Life Science, Okayama University, Okayama 700–8530, Japan; 3 Research and Technology Transfer Center, Nong Lam University, Ho Chi Minh City 700000, Vietnam

**Keywords:** Dairy cows, healthy udder, Illumina MiSeq, milk microbiota, somatic cell count, subclinical mastitis

## Abstract

**Objectives:** This study aimed to characterize and compare the milk microbiota composition among Holstein Friesian cows with healthy (HU), mastitis-suspected (MSU), and subclinical mastitis (SM) udders in Southern Vietnam.

**Materials and Methods:** Sixty milk samples were collected from two dairy farms and classified based on somatic cell counts (SCC) into three groups: HU (<200,000 cells/ml), MSU (200,000–400,000 cells/ml), and SM (>400,000 cells/ml). Bacterial communities were profiled using Illumina MiSeq sequencing of the *16S rRNA* gene.

**Results:** The prevalence of subclinical mastitis was 46.67% (*p* < 0.05). The core microbiome was dominated by phyla of *Proteobacteria, Firmicutes, Actinobacteria*, and *Bacteroidetes* (>90%); and taxa of *Moraxellaceae, Sphingomonadaceae, Enterobacteriaceae, Erysipelotrichaceae*, and *Streptococcaceae*. Analysis revealed significant dysbiosis in the SM group, characterized by elevated relative abundances of mastitis-associated taxa (*Mycobacteriaceae, Streptococcaceae, Moraxellaceae, Pasteurellaceae*, and *Mycoplasmataceae*) compared to healthy udders (*p* < 0.05). Conversely, commensal taxa typical of healthy milk (*Rikenellaceae, Lactobacillaceae, Sphingomonadaceae*, and *Opitutaceae*) were significantly depleted in SM samples but remained abundant in both HU and MSU groups (*p* < 0.05). Notably, the microbial profile of the MSU group was statistically similar to that of the HU group (*p* > 0.05), with no distinct variation in key bacterial families. Principal coordinate analysis further confirmed that SM samples formed a distinct cluster separate from the HU and MSU groups.

**Conclusions:** Subclinical mastitis drives significant shifts in the milk microbiome. However, mastitis-suspected cows retain a microbiome similar to that of healthy udders, suggesting that SCC thresholds alone may require careful interpretation in borderline cases.

## 1. Introduction

Mastitis has been one of the most common diseases in dairy cows, altering the physical, chemical, pathological, and bacterial properties of milk and mammary gland tissue [1]. It has been demonstrated that mastitis significantly reduces milk yield (>15%) and quality parameters, thereby affecting productivity and profitability in the dairy cow industry, especially in subclinical and clinical forms of this disease [2, 3, 4, 5, 6, 7, 8]. In previous investigation, there was a high prevalance of subclinical mastitis (a range of 30–57%) from dairy cows herd in Ho Chi Minh City, Vietnam, depending on the time of milking (days in milk) from 7 to 90 days after calving [5], whereas it was also reported that higher rates of the subclinical mastitis (63.2–88.6%) in Southern Vietnam [9]. Mastitis, manifesting in both clinical and subclinical forms, poses a major challenge for the dairy industry, resulting in significant economic losses from reduced milk yield, compromised milk quality, and increased veterinary costs [10, 11]. In particular, there are significant negative effects of milk from dairy cows with mastitis on consumer health [12, 13, 14], due to bacteria and their toxins, or to antibiotic residues in milk following antibiotic treatment [15, 16].

Recently, it has been indicated that there are many potential factors affecting the mastitis levels of postpartum dairy cows in Vietnam, including bacterial infections, pathogenic microorganisms, house conditions, climate, caring activities, milking hygiene, herd management, barn hygiene, feed, nutrition, and negative energy balance [4, 5, 17, 18]. These factors collectively contribute to dairy cows’ susceptibility to intramammary infections. Notably, subclinical mastitis can also progress to clinical cases, significantly affecting farmers’ economic efficiency. Factors related to pathogenic microorganisms causing mastitis in dairy cows include bacteria, bacterial toxins, mycoplasmas, and fungi [19]. Among these agents, *Staphylococcus* spp. is a major cause of subclinical and clinical mastitis in dairy cows, while *Staphylococcus aureus* and *Escherichia coli* are commonly isolated from the milk of dairy animals with clinical mastitis [20]. Crucially, the teat skin serves as a primary ecological niche and a significant reservoir for the milk microbiota. Recent studies have highlighted that the teat skin harbors diverse bacterial communities, including *Staphylococcus, Acinetobacter*, and *Aerococcus*, which can ascend through the teat canal to colonize the mammary gland or contaminate milk during milking. Therefore, dysbiosis of the teat skin microbiota is closely linked to intramammary infections and udder health status [21, 22]. Indeed, over 135 bacterial species and 20 common pathogens have been detected in cows with mastitis [21, 22]. While Ali et al. [23] found that *Staphylococcus* spp., *E. coli, Streptococcus* spp., and *Klebsiella* spp. were the most predominant bacterial agents isolated from mastitis milk, relying solely on the isolation of specific pathogens may not capture the full complexity of the disease. Therefore, understanding the comprehensive profile of potential pathogenic microorganisms and the broader microbiota is essential for effective treatment and prevention strategies.

The main goals of sustainable development of the dairy industry in Vietnam have been identified as increasing milk production, milk quality, and dairy herd fertility, as well as ensuring the food safety of dairy products for humans [9, 24, 25]. Effective prevention and treatment of mastitis are very important for achieving the above goal. Evaluating differences in milk microbiota between dairy cows with subclinical mastitis and healthy cows provides an important basis for assessing the epidemiological situation of subclinical mastitis, especially the bacteria most commonly associated with it. It is well known that the prevalence of subclinical mastitis has varied significantly across dairy farms, and the predominant mastitis pathogens have also differed considerably [26, 27]. In Vietnam, however, changes in the microbiota of milk from subclinical mastitis and healthy cows remain limited, and further studies are needed to clarify this point.

Therefore, the objective of this study was to determine differences in milk microbiota between subclinical mastitis and healthy cows on dairy farms in Vietnam, under Vietnam’s weather conditions, to help prevent mastitis and improve milk productivity/quality for the sustainable development of the dairy industry in Vietnam.

## 2. Materials and Methods

### 2.1. Ethical approval

The standard milk sampling procedure from dairy cows in this investigation was approved by the Animal Ethics Committees of Nong Lam University, Ho Chi Minh City, Vietnam (AEC-NLU).

### 2.2. Study area, animal selection, and sampling

The study was conducted at two commercial dairy farms in Ho Chi Minh City, Southern Vietnam. Farm 1, located in Binh Chanh District, maintained a total herd of 310 Holstein Friesian (HF) crossbreds, including 127 reproductive cows and 85 lactating cows, with an average milk yield of 20.15 kg/day/cow. The herd was managed using AfiFarm software (Afimilk Ltd., Israel). Farm 2, located in Cu Chi District, consisted of 120 HF crossbreds, including 62 reproductive cows and 41 lactating cows, with an average milk yield of 12.55 kg/day/cow. This farm utilized the Epacific management system (Epacific Informatics System JSC, Vietnam). The cows were housed in a free-stall barn with free access to water (*ad libitum*) and fed twice a day (around 7:45 and 14:15 every day, *ad libitum*) as a total mixed ration method (TMR) (Table 1).

**Table 1.** Ingredients in TMR rations at two farms (% on dry matter).

Ingredients	Unit	Farm 1	Farm 2
King grass	(%)	21.68	16.35
Alfafa grass	(%)	1.41	1.50
Mulato grass	(%)	5.88	0.00
Corn silage	(%)	36.12	13.65
Corn tree	(%)	0.00	25.00
Corn powder	(%)	3.06	3.60
Rice bran	(%)	0.47	0.00
Soybean meal	(%)	1.65	4.00
Complete powder	(%)	26.82	25.80
Rumifat flus	(%)	1.10	0.80
Brewers’ grain	(%)	1.68	7.80
Molasses	(%)	0.00	1.25
Vitamins and minerals	(%)	0.15	0.25
Total	(%)	100.00	100.00

Cow-level characteristics, including parity and days in milk (DIM), were extracted directly from the respective herd management records of each farm. Additionally, the body condition score (BCS) of each cow was evaluated by a single experienced veterinarian at the time of sampling to ensure consistency. The BCS was recorded using a standard 5-point scale [28, 29] with 0.25 increments, where scores of 1–2 represent emaciated and scores of 4–5 represent obese.

A total of 60 lactating cows (30 per farm) were selected for the study based on days in milk (DIM), parity, and body condition score (BCS) (Table 2). A total of 60 milk samples were collected individually from HF dairy cows at two farms in Southern Vietnam (30 per farm). Cows currently receiving antibiotic treatment or showing symptoms of clinical mastitis were strictly excluded from the study. Collecting raw data and milk samples as a cross-sectional study model at two farms.

**Table 2.** Characteristics of the dairy cow groups selected for the study based on Somatic Cell Count (SCC).

Farm	Groups	Cows per group (N)	% each group	SCC (× 10^3^ cells/ml)	DIM (days)	Parity	BCS
1 (30 cows)	HU	14	46.67^a^	107.8 ± 34.96^b^	46.80 ± 38.40	1.93 ± 0.73	2.80 ± 0.36
	MSU	3	10.00^b^	275.3 ± 39.0^b^	37.70 ± 27.00	1.67 ± 0.58	2.58 ± 0.14
	SM	13	43.33^a^	1,169 ± 442.00^a^	51.30 ± 40.90	2.15 ± 0.89	2.75 ± 0.27
	*p*		0.004	<0.01	0.853	0.580	0.535
2 (30 cows)	HU	9	30.00^ab^	131.7 ± 41.1^b^	38.60 ± 36.50	1.22 ± 0.44	2.94 ± 0.30
	MSU	6	20.00^b^	306.5 ± 52.2^b^	80.50 ± 35.00	1.00 ± 0.00	3.15 ± 0.41
	SM	15	50.00^a^	1,130 ± 444.00^a^	54.53 ± 35.27	1.07 ± 0.26	3.10 ± 0.49
	*p*		0.043	<0.01	0.101	0.333	0.604
Average (60 cows)	HU	23	38.33^a^	117.2 ± 38.43^b^	43.57 ± 37.02	1.65 ± 0.71	2.86 ± 0.34
	MSU	9	15.00^b^	296.1 ± 48.20^b^	66.20 ± 37.50	1.22 ± 0.44	2.96 ± 0.43
	SM	28	46.67^a^	1,148 ± 435.2^a^	53.04 ± 37.29	1.57 ± 0.84	2.94 ± 0.43
	*p*		<0.01	<0.01	0.294	0.340	0.729

Different superscripts indicate signifcant differences within within each farm in the same column (*p* < 0.05). HU, healthy udder with SCC criteria < 200,000 cells/ml; MSU, mastitis-suspected udder with SCC criteria from 200,000 to 400.000 cells/ml; SM, subclinical mastitis with SCC criteria > 400.000 cells/ml; DIM, days in milk; BCS, body condition score.

At both farms, each milk sample was collected after cleaning the surface of the udder, and the foremilk was discarded before collecting the sample. Milk samples were manually collected from 4 udders, then combined to produce a composite sample [4]. About 50 ml of milk was collected at milking time in the morning, transported quickly to a laboratory at 2–6°C, and stored at –20°C until further analyses. The SCC of the milk was determined using a CombiFoss FT^+^ analyzer (Foss Allé, Hillerød, Denmark).

### 2.3. Milk assessment for the prevalence of subclinical mastitis in dairy cows

Milk samples were individually collected and analyzed for mastitic disease through somatic cell count (SCC) level by Foss–Milkoscan machines in the milk center of the farms. Subclinical mastitis has been defined by an SCC level greater than 400,000 cells per milliliter of milk [6, 9, 30, 31, 32]. Based on Somatic Cell Count (SCC) analysis, the experimental animals were divided into three groups to evaluate the relationship between udder health and milk microbiota. The healthy udder (HU) group consisted of cows with an SCC < 200,000 cells/ml. The mastitis-suspected (MSU) group included cows with SCC ranging from 200,000 to 400,000 cells/ml, indicating an intermediate level of health. The subclinical mastitis (SM) group comprised cows with an SCC > 400,000 cells/ml, indicative of an intramammary infection without visible clinical signs.

### 2.4. Quantification of milk microbiota

#### 2.4.1. DNA extraction

The milk samples (250 μl per sample) were centrifuged at 16,000 × *g* for 2 min at room temperature, and the precipitate was collected for DNA isolation. Then, bacterial DNA from milk was extracted using the DNeasy Blood & Tissue Kit (Qiagen, Germantown, MD, USA) according to the manufacturer’s instructions. Crucially, to monitor for potential reagent contamination, a negative extraction control (250 μl of sterile nuclease-free water) was processed in parallel with each batch of samples. To minimize environmental contamination, all procedures were conducted in a dedicated pre-PCR facility using strict aseptic techniques and aerosol-resistant pipette tips.

#### 2.4.2. Illumina MiSeq sequencing

*16S rRNA* gene amplicon sequencing: Quantification of microbiota in milk samples was performed using bacterial DNA via polymerase chain reaction (PCR) according to a protocol previously described by one of our study members [4]. A no-template control (NTC) was included in all PCR runs to verify the absence of contamination. In brief, the two-step PCR was used to amplify bacterial DNA in order to create amplicon libraries for next-generation sequencing. The first round of PCR used specific primers to the V4 region of the bacterial *16S rRNA* genes (forward: 5′-ACA CTC TTC CCT ACA CGA CGC TCT TCC GAT CTG TGC CAG CMG CCG CGG TAA-3′; reverse: 5′-GTG ACT GGA GTT CAG ACG TGT GCT CTT CCG ATC TGG ACT ACH VGG GTW TCT AAT-3′). The procedure of the first round of the PCR included initial denaturation (94°C for 2 min), followed by 35 cycles of denaturation (94°C for 30 sec), annealing (50°C for 30 sec), elongation (72°C for 30 sec), and a last step at an elongation (72°C for 5 min). PCR products were visualized via electrophoresis on a 2.0% agarose gel using the FastGene Gel/PCR extraction kit (Nippon Genetics Co., Ltd., Tokyo, Japan); no bands were observed in the negative controls, confirming the integrity of the process. Next, the second round of PCR (combined with adapter-attached primers) with standard protocol included initial denaturation (94°C for 2 min), followed by 10 cycles of denaturation (94°C for 30 sec), annealing (59°C for 30 sec), elongation (72°C for 30 sec), and a final step at an elongation (72°C for 5 min). The purification process for the products at the second-round PCR step was identical to that used for the first-round PCR products [4].

The Illumina MiSeq platform at FASMAC Co., Ltd. (Kanagawa, Japan) was used to pair-end sequence the purified amplicons (2 × 250 bp). The Quantitative Insights Into Microbial Ecology (QIIME version 1.9.0) was used to analyze the raw sequencing data. Any site with an average quality score below 20 had the 250-bp reads truncated; of course, the truncated reads were discarded (no shorter than 225 bp). A threshold of 97% similarity was used to classify the final pair-end joining reads into operational taxonomic units. The Ribosomal Database Project classifier›s default parameters were used to evaluate and classify the sequencing data at the phylum to family level [4].

### 2.5. Statistical analysis

Data were collected and analyzed using GraphPad Prism 8.3 (GraphPad Software, Inc., San Diego, CA, USA). Quantitative data on general information for subgroups of dairy cows were evaluated using one-way analysis of variance (ANOVA) followed by Tukey’s multiple-range post hoc test, while qualitative values (%) were compared using the χ^2^ test. The data on the relative abundances of the major bacterial phyla and families (families with >1.0%) were analyzed using the nonparametric Kruskal-Wallis or Mann-Whitney U test for three or two groups of cattle, respectively, to examine the effect of dairy udder health status on the milk microbiota. The bacterial data were also subjected to canonical analysis of principal coordinates (CAPC) to determine assignments and clustering, thereby elucidating microbiota variation. Discriminant vectors were considered significant if the Pearson correlation was > 0.7. One-way ANOVA followed by the Tukey test was performed using JMP (version 11; SAS Institute, Tokyo, Japan), and CAP was performed using Primer version 7 with the Permanova^+^ add-on (Primer-E, Plymouth Marine Laboratory, Plymouth, UK). In these analyses, statistically significant differences were considered at *p* < 0.05.

## 3. Results

### 3.1. The mastitis prevalence and general information of groups of dairy cows

As shown in Table 2, the subclinical mastitis group accounted for the highest proportion of the herd (46.67%). This prevalence was significantly greater than that in mastitis-suspected cows (15.00%; *p* < 0.05), though it remained statistically comparable to that in the healthy udder group (38.33%; *p* > 0.05). Regarding the somatic cell count (SCC), a clear distinction was observed; the subclinical mastitis group exhibited a dramatically elevated mean SCC of 1,148 × 10^3^ cells/ml. This value was significantly higher (*p* < 0.01) compared to both the healthy (117.2 × 10^3^ cells/ml) and mastitis-suspected (296.1 × 10^3^ cells/ml) groups. Furthermore, no significant differences (*p* > 0.05; Table 2) were detected in DIM, parity, and BCS among the three groups at different stages of mastitis.

### 3.2. Microbiota of milk from dairy cows with different statuses of udder health

**Figure 1.**
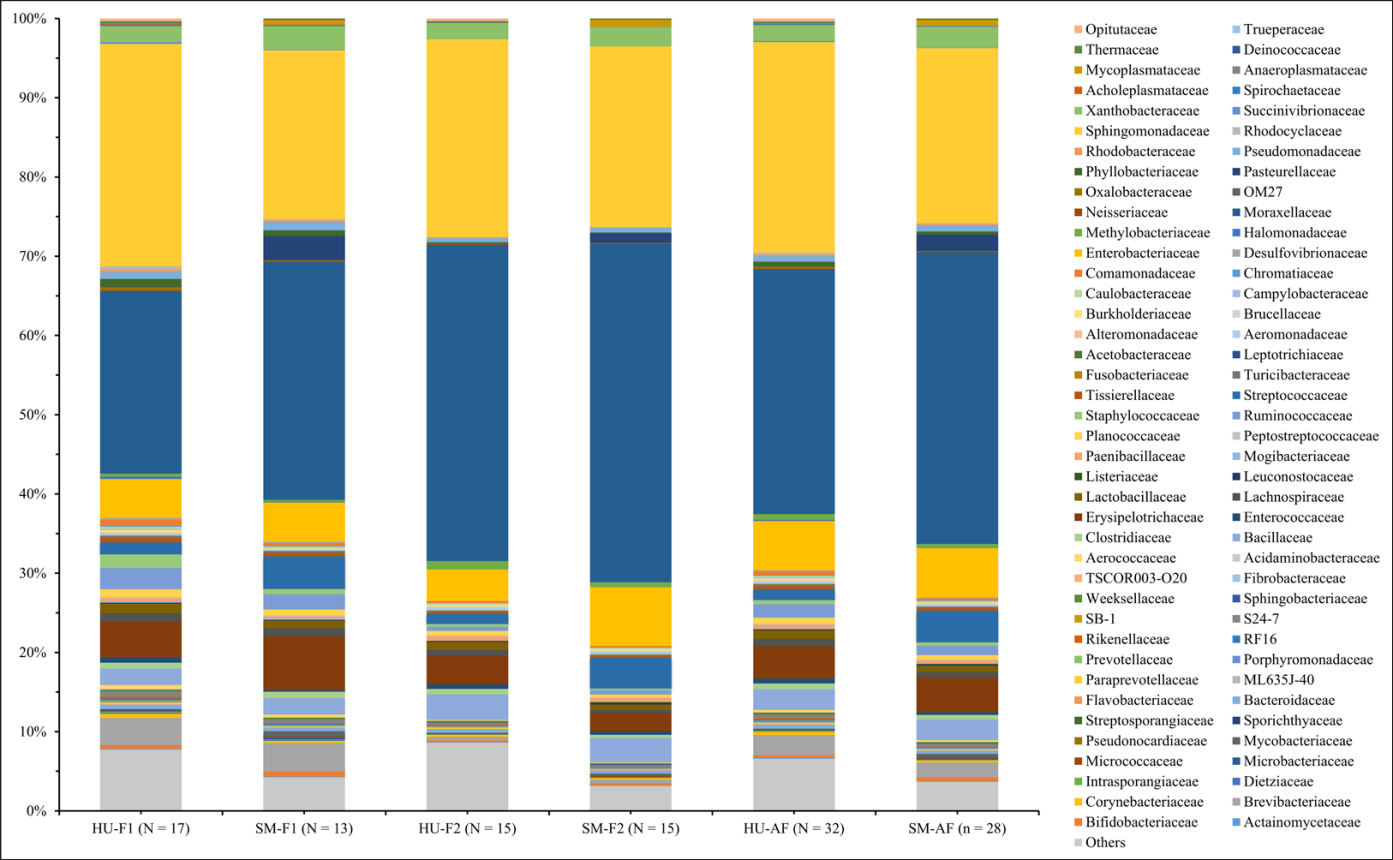
Milk microbiota from two groups of dairy cows with different health statuses of the udder.

**Figure 2.**
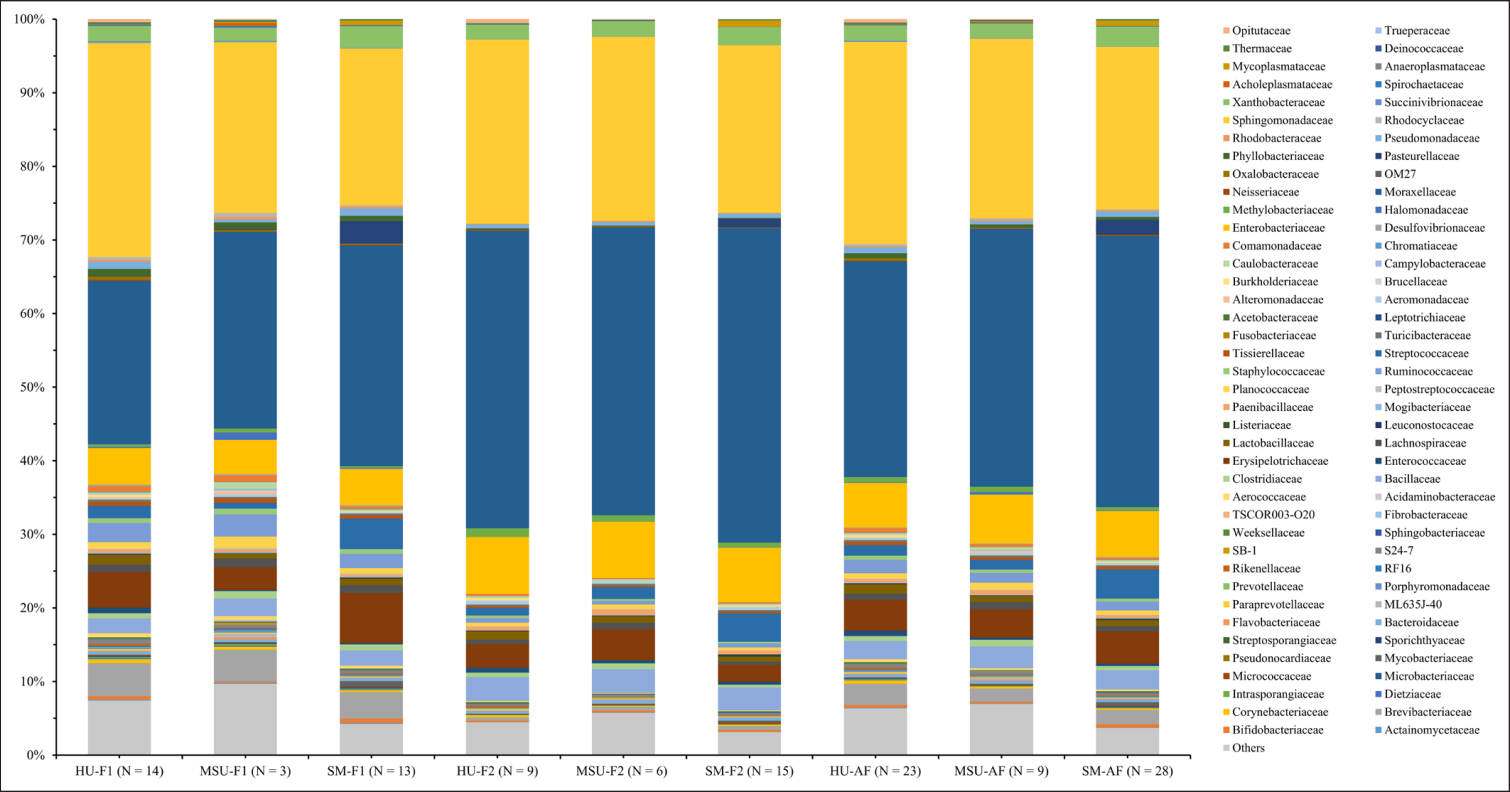
Milk microbiota from three groups of dairy cows with different health statuses of the udder.

The Illumina MiSeq sequencing results indicated that the milk microbiota showed high taxonomic diversity, including 37 phyla and 189 families, of which 13 phyla and 80 families were shared across all samples (Figures 1, 2). There was a majority of the four main phyla from *Proteobacteria, Firmicutes, Actinobacteria*, and *Bacteroidetes*, which accounted for > 90% of the total milk microbiota, regardless of each farm or all farms. Overall, the abundance of *Proteobacteria* was significantly higher than the total of *Firmicutes, Actinobacteria*, and *Bacteroidetes*. Interestingly, there were significant differences (*p* > 0.05) in these four major phyla between the healthy udder and subclinical mastitis groups (Figure 1) or among the three groups of cows (healthy udder, mastitis-suspected udder, and subclinical mastitis; Figure 2). In contrast, the abundances of *Tenericutes* were significantly increased (*p* < 0.05), and those of *Verrucomicrobia* were remarkably decreased (*p* < 0.05) in milk with the mastitis-suspected udder and/or subclinical mastitis as compared to those of healthy udder groups (Figures 1, 2) at each farm or all farms.

Compositional differences of milk microbiota from two groups of dairy cows were significantly found (*p* < 0.05; Table 3) in some phyla/families in the milk sample at each farm or the average of two farms. The five most common families found in the milk microbiota from healthy udder or subclinical mastitis groups of dairy cows were *Moraxellaceae* (average 30.96 or 36.87%, respectively), *Sphingomonadaceae* (average 26.68 or 22.17%, respectively), *Enterobacteriaceae* (average 6.23 or 6.28%, respectively), *Erysipelotrichaceae* (average 4.10 or 4.38%, respectively), and *Streptococcaceae* (average 1.39 or 3.98%, respectively). The compositional differences of milk microbiota from two groups of dairy cows were seen for some specific families at the average of two farms; relative abundances of *Mycobacteriaceae* (0.01%), *Streptococcaceae* (1.39%), *Moraxellaceae* (30.96%), *Pasteurellaceae* (0.02%) and *Mycoplasmataceae* (0.05%) in milk from healthy udder were significantly lower (*p* < 0.05) than those of subclinical mastitis groups (0.41, 3.98, 36.87, 2.04, and 0.77, respectively); while relative ones of *Sphingomonadaceae* (26.68%) and *Opitutaceae* (0.35%) in milk from healthy udder were significantly higher (*p* < 0.05) than those of subclinical mastitis groups (22.17 and 0.01%, respectively). Of course, these significant changes (*p* < 0.05) were also similar across the two farms (1 and 2). The other compositions of milk microbiota, however, were not significantly varied (*p* > 0.05; Table 3) between the healthy udder and subclinical mastitis at farms 1, 2, and the average of the two farms.

**Table 3.** Relative abundance (%) of milk microbiota from two groups of dairy cows with different udder health statuses.

Farm	1		2		Average	
Phylum/Family	HU (N = 17)	SM (N = 13)	HU (N = 15)	SM (N = 15)	HU (N = 32)	SM (N = 28)
*Acidobacteria*	0.04 ± 0.14	0.00 ± 0.00	0.01 ± 0.04	0.01 ± 0.01	0.03 ± 0.10	0.01 ± 0.01
*Actinobacteria*	6.40 ± 5.98	6.27 ± 5.22	1.44 ± 0.88	1.55 ± 0.97	4.07 ± 5.01	3.74 ± 4.29
*Actainomycetaceae*	0.13 ± 0.13	0.10 ± 0.11	0.04 ± 0.06	0.02 ± 0.02	0.09 ± 0.11	0.06 ± 0.08
*Bifidobacteriaceae*	0.44 ± 0.33	0.63 ± 0.89	0.22 ± 0.42	0.29 ± 0.41	0.34 ± 0.39	0.45 ± 0.69
*Brevibacteriaceae*	4.44 ± 5.59	3.57 ± 4.98	0.46 ± 0.36	0.49 ± 0.5	2.57 ± 4.50	1.92 ± 3.68
*Corynebacteriaceae*	0.52 ± 0.63	0.28 ± 0.23	0.27 ± 0.25	0.21 ± 0.17	0.40 ± 0.50	0.24 ± 0.20
*Dietziaceae*	0.12 ± 0.12	0.11 ± 0.14	0.00 ± 0.00	0.01 ± 0.01	0.06 ± 0.11	0.05 ± 0.10
*Intrasporangiaceae*	0.16 ± 0.14	0.10 ± 0.10	0.02 ± 0.05	0.05 ± 0.04	0.10 ± 0.13	0.07 ± 0.07
*Microbacteriaceae*	0.13 ± 0.14	0.19 ± 0.19	0.07 ± 0.08	0.05 ± 0.06	0.10 ± 0.12	0.12 ± 0.15
*Micrococcaceae*	0.13 ± 0.10	0.14 ± 0.07	0.10 ± 0.06	0.20 ± 0.29	0.11 ± 0.09	0.17 ± 0.22
*Mycobacteriacea*e	0.01 ± 0.02^b^	0.67 ± 0.16^a^	0.01 ± 0.02^b^	0.20 ± 0.09^a^	0.01 ± 0.02^b^	0.41 ± 0.07^a^
*Pseudonocardiaceae*	0.01 ± 0.02	0.00 ± 0.00	0.01 ± 0.03	0.01 ± 0.01	0.01 ± 0.02	0.00 ± 0.00
*Sporichthyaceae*	0.01 ± 0.01	0.00 ± 0.00	0.01 ± 0.02	0.00 ± 0.00	0.01 ± 0.02	0.00 ± 0.00
*Streptosporangiaceae*	0.00 ± 0.00	0.01 ± 0.02	0.00 ± 0.00	0.01 ± 0.01	0.00 ± 0.00	0.01 ± 0.01
*Bacteroidetes*	2.67 ± 1.45	1.83 ± 1.24	1.59 ± 0.83	1.39 ± 0.42	2.16 ± 1.30	1.60 ± 0.91
*Bacteroidaceae*	0.45 ± 0.25	0.40 ± 0.26	0.45 ± 0.21	0.42 ± 0.21	0.44 ± 0.22	0.41 ± 0.23
*Flavobacteriaceae*	0.12 ± 0.16	0.06 ± 0.10	0.06 ± 0.09	0.06 ± 0.07	0.09 ± 0.13	0.06 ± 0.08
*ML635J-40*	0.10 ± 0.33	0.03 ± 0.06	0.00 ± 0.00	0.00 ± 0.00	0.05 ± 0.24	0.01 ± 0.02
*Paraprevotellaceae*	0.21 ± 0.16	0.14 ± 0.15	0.17 ± 0.14	0.12 ± 0.11	0.19 ± 0.15	0.13 ± 0.13
*Porphyromonadaceae*	0.18 ± 0.16	0.12 ± 0.13	0.10 ± 0.12	0.05 ± 0.09	0.14 ± 0.14	0.09 ± 0.11
*Prevotellaceae*	0.09 ± 0.09	0.05 ± 0.06	0.06 ± 0.10	0.02 ± 0.05	0.08 ± 0.09	0.04 ± 0.05
*RF16*	0.20 ± 0.18	0.13 ± 0.17	0.03 ± 0.07	0.02 ± 0.04	0.12 ± 0.16	0.06 ± 0.13
*Rikenellaceae*	0.22 ± 0.21	0.10 ± 0.04	0.17 ± 0.20	0.09 ± 0.14	0.20 ± 0.10	0.09 ± 0.03
*S24–7*	0.63 ± 0.43	0.47 ± 0.48	0.27 ± 0.18	0.30 ± 0.24	0.46 ± 0.38	0.38 ± 0.37
*SB-1*	0.05 ± 0.14	0.01 ± 0.02	0.00 ± 0.00	0.00 ± 0.00	0.03 ± 0.10	0.01 ± 0.01
*Sphingobacteriaceae*	0.05 ± 0.06	0.05 ± 0.06	0.06 ± 0.08	0.11 ± 0.10	0.05 ± 0.07	0.08 ± 0.15
*Weeksellaceae*	0.18 ± 0.29	0.16 ± 0.16	0.15 ± 0.27	0.12 ± 0.11	0.17 ± 0.28	0.14 ± 0.13
*Chlamydiae*	0.01 ± 0.04	0.04 ± 0.09	0.01 ± 0.02	0.06 ± 0.14	0.01 ± 0.03	0.05 ± 0.12
*Euryarchaeota*	0.08 ± 0.07	0.07 ± 0.08	0.04 ± 0.08	0.02 ± 0.02	0.06 ± 0.08	0.04 ± 0.06
*Fibrobacteres*	0.05 ± 0.07	0.00 ± 0.00	0.01 ± 0.02	0.00 ± 0.00	0.02 ± 0.05	0.00 ± 0.00
*Fibrobacteraceae*	0.02 ± 0.04	0.00 ± 0.00	0.01 ± 0.02	0.00 ± 0.00	0.01 ± 0.03	0.00 ± 0.00
*TSCOR003-O20*	0.01 ± 0.04	0.00 ± 0.00	0.00 ± 0.00	0.00 ± 0.00	0.01 ± 0.02	0.00 ± 0.00
*Firmicutes*	18.51 ± 4.44	21.23 ± 12.06	14.09 ± 9.81	14.45 ± 5.87	16.44 ± 7.66	17.60 ± 9.71
*Acidaminobacteraceae*	0.05 ± 0.10	0.01 ± 0.04	0.00 ± 0.00	0.01 ± 0.03	0.02 ± 0.07	0.01 ± 0.03
*Aerococcaceae*	0.50 ± 0.49	0.37 ± 0.48	0.12 ± 0.17	0.08 ± 0.10	0.32 ± 0.42	0.21 ± 0.36
*Bacillacea*e	2.06 ± 1.19	2.08 ± 1.34	3.21 ± 0.86	3.11 ± 1.06	2.60 ± 1.18	2.62 ± 1.29
*Clostridiaceae*	0.75 ± 0.67	0.81 ± 0.81	0.71 ± 0.79	0.39 ± 0.42	0.73 ± 0.72	0.58 ± 0.65
*Enterococcaceae*	0.67 ± 1.41	0.21 ± 0.22	0.56 ± 0.58	0.43 ± 0.15	0.62 ± 1.08	0.33 ± 0.21
*Erysipelotrichacea*e	4.53 ± 4.42	6.79 ± 10.13	3.62 ± 7.06	2.29 ± 3.56	4.10 ± 5.73	4.38 ± 7.58
*Lachnospiraceae*	1.03 ± 0.44	1.05 ± 0.50	0.72 ± 0.58	0.42 ± 0.23	0.88 ± 0.53	0.71 ± 0.49
*Lactobacillaceae*	1.24 ± 1.15	0.89 ± 0.26	1.02 ± 0.53	0.69 ± 0.30	1.13 ± 0.90	0.79 ± 0.39
*Leuconostocaceae*	0.10 ± 0.11	0.08 ± 0.11	0.11 ± 0.06	0.17 ± 0.1	0.10 ± 0.09	0.13 ± 0.12
*Listeriaceae*	0.00 ± 0.00	0.07 ± 0.15	0.00 ± 0.00	0.13 ± 0.26	0.00 ± 0.00	0.10 ± 0.12
*Mogibacteriaceae*	0.14 ± 0.12	0.11 ± 0.14	0.02 ± 0.03	0.01 ± 0.03	0.08 ± 0.11	0.06 ± 0.10
*Paenibacillaceae*	0.43 ± 0.42	0.27 ± 0.30	0.61 ± 0.25	0.51 ± 0.27	0.52 ± 0.36	0.40 ± 0.31
*Peptostreptococcaceae*	0.12 ± 0.15	0.13 ± 0.22	0.08 ± 0.07	0.04 ± 0.08	0.09 ± 0.12	0.08 ± 0.16
*Planococcaceae*	1.01 ± 0.90	0.78 ± 0.29	0.56 ± 0.35	0.38 ± 0.24	0.79 ± 0.73	0.57 ± 0.33
*Ruminococcaceae*	2.68 ± 2.29	1.91 ± 2.40	0.54 ± 0.40	0.54 ± 0.39	1.67 ± 1.98	1.18 ± 1.77
*Staphylococcaceae*	1.72 ± 1.23	0.67 ± 0.62	0.34 ± 0.32	0.23 ± 0.17	0.52 ± 0.40	0.43 ± 0.48
*Streptococcacea*e	1.53 ± 1.08^b^	4.13 ± 1.79^a^	1.25 ± 1.78^b^	3.86 ± 1.69^a^	1.39 ± 1.44^b^	3.98 ± 4.21^a^
*Tissierellaceae*	0.54 ± 0.39	0.44 ± 0.37	0.23 ± 0.21	0.25 ± 0.23	0.39 ± 0.35	0.34 ± 0.31
*Turicibacteraceae*	0.25 ± 0.17	0.20 ± 0.16	0.16 ± 0.14	0.17 ± 0.18	0.21 ± 0.16	0.18 ± 0.17
*Fusobacteria*	0.05 ± 0.07	0.01 ± 0.01	0.05 ± 0.07	0.03 ± 0.04	0.05 ± 0.07	0.02 ± 0.03
*Fusobacteriaceae*	0.05 ± 0.07	0.00 ± 0.00	0.05 ± 0.07	0.03 ± 0.04	0.05 ± 0.07	0.02 ± 0.03
*Leptotrichiaceae*	0.00 ± 0.00	0.01 ± 0.01	0.00 ± 0.00	0.00 ± 0.00	0.00 ± 0.00	0.00 ± 0.00
*Proteobacteria*	64.68 ± 7.44	66.98 ± 14.25	79.78 ± 10.82	80.75 ± 6.43	71.76 ± 11.83	74.36 ± 12.67
*Acetobacteraceae*	0.02 ± 0.05	0.02 ± 0.06	0.02 ± 0.04	0.01 ± 0.02	0.02 ± 0.05	0.01 ± 0.05
*Aeromonadaceae*	0.21 ± 0.20	0.17 ± 0.16	0.43 ± 0.15	0.38 ± 0.16	0.32 ± 0.21	0.28 ± 0.19
*Alteromonadaceae*	0.15 ± 0.39	0.02 ± 0.09	0.00 ± 0.00	0.01 ± 0.04	0.06 ± 0.28	0.02 ± 0.04
*Brucellaceae*	0.02 ± 0.03	0.01 ± 0.01	0.03 ± 0.04	0.03 ± 0.04	0.02 ± 0.03	0.02 ± 0.03
*Burkholderiaceae*	0.32 ± 0.65	0.11 ± 0.13	0.22 ± 0.40	0.23 ± 0.17	0.27 ± 0.54	0.17 ± 0.16
*Campylobacteraceae*	0.11 ± 0.20	0.02 ± 0.05	0.00 ± 0.00	0.01 ± 0.02	0.06 ± 0.16	0.01 ± 0.03
*Caulobacteraceae*	0.30 ± 0.59	0.23 ± 0.37	0.24 ± 0.24	0.18 ± 0.19	0.27 ± 0.45	0.20 ± 0.28
*Chromatiaceae*	0.11 ± 0.12	0.10 ± 0.10	0.02 ± 0.05	0.04 ± 0.12	0.07 ± 0.10	0.07 ± 0.11
*Comamonadaceae*	0.78 ± 1.06	0.32 ± 0.16	0.23 ± 0.15	0.17 ± 0.15	0.52 ± 0.81	0.24 ± 0.17
*Desulfovibrionaceae*	0.20 ± 0.23	0.17 ± 0.26	0.01 ± 0.02	0.01 ± 0.05	0.11 ± 0.19	0.09 ± 0.19
*Enterobacteriacea*e	4.92 ± 3.53	4.96 ± 3.25	3.99 ± 7.41	7.42 ± 1.12	6.23 ± 3.07	6.28 ± 2.63
*Halomonadaceae*	0.29 ± 0.65	0.13 ± 0.11	0.01 ± 0.01	0.02 ± 0.03	0.15 ± 0.48	0.07 ± 0.10
*Methylobacteriaceae*	0.40 ± 0.32	0.22 ± 0.11	1.06 ± 0.70	0.64 ± 0.55	0.71 ± 0.62	0.44 ± 0.45
*Moraxellacea*e	23.02 ± 5.52^b^	30.09 ± 4.87^a^	39.96 ± 7.41	42.74 ± 3.95	30.96 ± 4.40^b^	36.87 ± 2.15^a^
*Neisseriaceae*	0.11 ± 0.16	0.09 ± 0.09	0.09 ± 0.13	0.06 ± 0.11	0.10 ± 0.14	0.08 ± 0.10
*OM27*	0.01 ± 0.01	0.00 ± 0.00	0.00 ± 0.00	0.00 ± 0.00	0.00 ± 0.00	0.00 ± 0.00
*Oxalobacteraceae*	0.41 ± 0.57	0.15 ± 0.21	0.07 ± 0.08	0.06 ± 0.07	0.25 ± 0.45	0.10 ± 0.16
*Pasteurellacea*e	0.02 ± 0.05^b^	3.05 ± 1.79^a^	0.01 ± 0.03^b^	1.17 ± 1.08^a^	0.02 ± 0.04^b^	2.04 ±1.30^a^
*Phyllobacteriaceae*	1.02 ± 1.06	0.72 ± 0.90	0.14 ± 0.13	0.14 ± 0.11	0.60 ± 0.89	0.41 ± 0.67
*Pseudomonadaceae*	0.86 ± 0.95	0.99 ± 1.31	0.52 ± 0.19	0.58 ± 0.21	0.70 ± 0.71	0.77 ± 0.91
*Rhodobacteraceae*	0.27 ± 0.22	0.24 ± 0.20	0.11 ± 0.10	0.09 ± 0.11	0.19 ± 0.19	0.16 ± 0.17
*Rhodocyclaceae*	0.42 ± 0.41	0.21 ± 0.26	0.01 ± 0.03	0.01 ± 0.02	0.23 ± 0.36	0.10 ± 0.20
*Sphingomonadacea*e	28.13 ± 3.29^a^	21.38 ± 7.18^b^	25.04 ± 2.19^b^	22.86 ± 2.6^a^	26.68 ± 9.40^a^	22.17 ± 2.34^b^
*Succinivibrionaceae*	0.22 ± 0.27	0.07 ± 0.10	0.02 ± 0.03	0.01 ± 0.03	0.12 ± 0.22	0.04 ± 0.07
*Xanthobacteraceae*	2.03 ± 0.53	2.95 ± 3.78	2.04 ± 0.44	2.44 ± 1.48	2.03 ± 0.48	2.68 ± 2.55
*Spirochaetes*	0.17 ± 0.18	0.14 ± 0.19	0.01 ± 0.03	0.02 ± 0.04	0.10 ± 0.15	0.08 ± 0.14
*Spirochaetaceae*	0.17 ± 0.18	0.14 ± 0.19	0.01 ± 0.03	0.02 ± 0.04	0.10 ± 0.15	0.08 ± 0.14
*Tenericutes*	0.19 ± 0.42^b^	0.63 ± 0.81^a^	0.02 ± 0.08^b^	0.91 ± 1.06^a^	0.11 ± 0.32^b^	0.78 ± 0.95^a^
*Acholeplasmataceae*	0.12 ± 0.32	0.03 ± 0.04	0.00 ± 0.00	0.00 ± 0.00	0.06 ± 0.24	0.01 ± 0.03
*Anaeroplasmataceae*	0.00 ± 0.00	0.00 ± 0.00	0.00 ± 0.00	0.00 ± 0.00	0.00 ± 0.00	0.00 ± 0.00
*Mycoplasmatacea*e	0.07 ± 0.11^b^	0.60 ± 0.80^a^	0.02 ± 0.08^b^	0.91 ± 1.06^a^	0.05 ± 0.10^b^	0.77 ± 0.95^a^
*Thermi*	0.27 ± 0.26	0.20 ± 0.09	0.17 ± 0.14	0.14 ± 0.10	0.22 ± 0.21	0.17 ± 0.10
*Deinococcaceae*	0.04 ± 0.04	0.05 ± 0.06	0.08 ± 0.12	0.05 ± 0.04	0.06 ± 0.08	0.05 ± 0.05
*Thermaceae*	0.21 ± 0.24	0.14 ± 0.12	0.08 ± 0.12	0.08 ± 0.08	0.15 ± 0.20	0.11 ± 0.10
*Trueperaceae*	0.01 ± 0.02	0.00 ± 0.00	0.01 ± 0.03	0.01 ± 0.02	0.05 ± 0.10	0.00 ± 0.00
*Verrucomicrobia*	0.41 ± 1.11	0.07 ± 0.08	0.47 ± 1.17^a^	0.01 ± 0.04^b^	0.44 ± 1.12^a^	0.04 ± 0.07^b^
*Opitutacea*e	0.34 ± 1.08	0.01 ± 0.04	0.35 ± 0.97^a^	0.01 ± 0.05^b^	0.35 ± 1.01^a^	0.01 ± 0.03^b^

Different superscripts indicate significant differences within each farm in the same row (*p* < 0.05). HU, healthy udder; SM, subclinical mastitis.

Otherwise, when it was divided into three groups of health status of cow udder based on the SCC findings (healthy udder, mastitis-suspected udder and subclinical mastitis), the results showed that compositional differences of milk microbiota from three groups of dairy cows were also signigitantly found (*p* < 0.05; Table 4) in some phyla/families in the milk sample at each farm or the average of two farms. *Moraxellaceae* (average 29.37, 35.01 or 36.87%, respectively), *Sphingomonadaceae* (average 27.54, 24.49 or 22.17%, respectively), *Enterobacteriaceae* (average 6.06, 6.66 or 6.28%, respectively), *Erysipelotrichaceae* (average 4.20, 3.85 or 4.38%, respectively), and *Streptococcaceae* (average 1.43, 1.33 or 3.98%, respectively) were also the five most common families found in the milk microbiota from healthy udder, mastitis-suspected udder or subclinical mastitis groups of dairy cows. The compositional differences of milk microbiota from three groups of dairy cows were seen for some specific families at the average of two farms; relative abundances of *Mycobacteriaceae* (0.01%), *Streptococcaceae* (1.43%), *Moraxellaceae* (29.37%), *Pasteurellaceae* (0.01%) and *Mycoplasmataceae* (0.03%) in milk from healthy udder were similar (*p* > 0.05) to those of mastitis-suspected udder (0.01, 1.33, 35.01, 0.03, and 0.09%, respectively) and significantly lower (*p* < 0.05) than those of subclinical mastitis groups (0.41, 3.98, 36.87, 2.04, and 0.77%, respectively); while relative ones of *Rikenellaceae* (0.23%), *Lactobacillaceae* (1.27%), *Sphingomonadaceae* (27.54%) and *Opitutaceae* (0.46%) in milk from healthy udder were similar (*p* > 0.05) to those of mastitis-suspected udder (0.10, 0.80, 24.49, and 0.07%, respectively) and were significantly higher (*p* < 0.05) those of subclinical mastitis groups (0.09, 0.79, 22.17, and 0.01%, respectively). Of course, these significant changes (*p* < 0.05) were also similar across the two farms (1 and 2). The other compositions of milk microbiota, however, were not significantly varied (*p* > 0.05; Table 4) among the healthy, mastitis-suspected udder and subclinical mastitis cows at farms 1, 2, and the average of the two farms.

**Table 4.** Compositional differences (%) of milk microbiota from three groups of dairy cows with different udder health statuses.

Farm	1			2			Average		
Phylum/Family	HU (N = 14)	MSU (N = 3)	SM (N = 13)	HU (N = 9)	MSU (N = 6)	SM (N = 15)	HU (N = 23)	MSU (N = 9)	SM (N = 28)
*Acidobacteria*	0.04 ± 0.15	0.00 ± 0.00	0.00 ± 0.00	0.02 ± 0.06	0.00 ± 0.00	0.01 ± 0.01	0.04 ± 0.12	0.00 ± 0.00	0.01 ± 0.01
*Actinobacteria*	6.44 ± 5.89	6.21 ± 7.78	6.27 ± 5.22	1.45 ± 0.79	1.43 ± 1.07	1.55 ± 0.97	4.49 ± 5.19	3.02 ± 4.64	3.74 ± 4.29
*Actainomycetaceae*	0.13 ± 0.13	0.11 ± 0.14	0.10 ± 0.11	0.04 ± 0.07	0.05 ± 0.03	0.02 ± 0.02	0.09 ± 0.12	0.07 ± 0.08	0.06 ± 0.08
*Bifidobacteriaceae*	0.49 ± 0.34	0.20 ± 0.03	0.63 ± 0.89	0.19 ± 0.25	0.27 ± 0.63	0.29 ± 0.41	0.37 ± 0.34	0.25 ± 0.50	0.45 ± 0.69
*Brevibacteriaceae*	4.47 ± 5.47	4.30 ± 7.42	3.57 ± 4.98	0.42 ± 0.38	0.52 ± 0.36	0.49 ± 0.5	2.88 ± 4.67	1.78 ± 4.17	1.92 ± 3.68
*Corynebacteriaceae*	0.54 ± 0.70	0.43 ± 0.20	0.28 ± 0.23	0.34 ± 0.29	0.15 ± 0.10	0.21 ± 0.17	0.46 ± 0.57	0.25 ± 0.19	0.24 ± 0.20
*Dietziaceae*	0.13 ± 0.13	0.10 ± 0.11	0.11 ± 0.14	0.00 ± 0.00	0.00 ± 0.00	0.01 ± 0.01	0.08 ± 0.12	0.03 ± 0.07	0.05 ± 0.10
*Intrasporangiaceae*	0.15 ± 0.14	0.24 ± 0.17	0.10 ± 0.10	0.01 ± 0.01	0.04 ± 0.07	0.05 ± 0.04	0.09 ± 0.12	0.11 ± 0.14	0.07 ± 0.07
*Microbacteriaceae*	0.13 ± 0.14	0.15 ± 0.16	0.19 ± 0.19	0.09 ± 0.10	0.03 ± 0.04	0.05 ± 0.06	0.12 ± 0.12	0.07 ± 0.10	0.12 ± 0.15
*Micrococcaceae*	0.14 ± 0.10	0.11 ± 0.13	0.14 ± 0.07	0.07 ± 0.05	0.13 ± 0.08	0.20 ± 0.29	0.11 ± 0.09	0.12 ± 0.09	0.17 ± 0.22
*Mycobacteriaceae*	0.01 ± 0.02^b^	0.01 ± 0.01^b^	0.67 ± 0.16^a^	0.00 ± 0.00^b^	0.02 ± 0.03^ab^	0.20 ± 0.09^a^	0.01 ± 0.01^b^	0.01 ± 0.03^b^	0.41 ± 0.07^a^
*Pseudonocardiaceae*	0.01 ± 0.02	0.01 ± 0.01	0.00 ± 0.00	0.00 ± 0.00	0.03 ± 0.04	0.01 ± 0.01	0.01 ± 0.01	0.02 ± 0.03	0.00 ± 0.00
*Sporichthyaceae*	0.01 ± 0.02	0.00 ± 0.00	0.00 ± 0.00	0.00 ± 0.00	0.01 ± 0.03	0.00 ± 0.00	0.00 ± 0.00	0.01 ± 0.02	0.00 ± 0.00
*Streptosporangiaceae*	0.00 ± 0.00	0.00 ± 0.00	0.01 ± 0.02	0.00 ± 0.00	0.00 ± 0.00	0.01 ± 0.01	0.00 ± 0.00	0.00 ± 0.00	0.01 ± 0.01
*Bacteroidetes*	2.52 ±1.25	3.38 ± 2.38	1.83 ± 1.24	1.69 ± 0.88	1.43 ± 0.82	1.39 ± 0.42	2.19 ± 1.17	2.08 ± 1.66	1.60 ± 0.91
*Bacteroidaceae*	0.47 ± 0.26	0.35 ± 0.17	0.40 ± 0.26	0.40 ± 0.15	0.51 ± 0.26	0.42 ± 0.21	0.44 ± 0.22	0.46 ± 0.23	0.41 ± 0.23
*Flavobacteriaceae*	0.08 ± 0.11	0.30 ± 0.26	0.06 ± 0.10	0.04 ± 0.06	0.09 ± 0.12	0.06 ± 0.07	0.06 ± 0.10	0.16 ± 0.19	0.06 ± 0.08
*ML635J-40*	0.02 ± 0.04	0.48 ± 0.79	0.03 ± 0.06	0.00 ± 0.00	0.00 ± 0.00	0.00 ± 0.00	0.01 ± 0.03	0.16 ± 0.46	0.01 ± 0.02
*Paraprevotellaceae*	0.22 ± 0.17	0.15 ± 0.14	0.14 ± 0.15	0.21 ± 0.15	0.10 ± 0.13	0.12 ± 0.11	0.22 ± 0.15	0.12 ± 0.12	0.13 ± 0.13
*Porphyromonadaceae*	0.16 ± 0.13	0.24 ± 0.27	0.12 ± 0.13	0.12 ± 0.15	0.07 ± 0.07	0.05 ± 0.09	0.14 ± 0.14	0.13 ± 0.17	0.09 ± 0.11
*Prevotellaceae*	0.10 ± 0.09	0.06 ± 0.10	0.05 ± 0.06	0.05 ± 0.09	0.08 ± 0.11	0.02 ± 0.05	0.08 ± 0.09	0.08 ± 0.10	0.04 ± 0.05
*RF16*	0.19 ± 0.18	0.25 ± 0.22	0.13 ± 0.17	0.05 ± 0.09	0.00 ± 0.00	0.02 ± 0.04	0.13 ± 0.16	0.08 ± 0.16	0.06 ± 0.13
*Rikenellacea*e	0.24 ± 0.02	0.12 ± 0.10	0.10 ± 0.04	0.22 ± 0.19	0.09 ± 0.20	0.09 ± 0.14	0.23 ± 0.03^a^	0.10 ± 0.11^ab^	0.09 ± 0.03^b^
*S24–7*	0.67 ± 0.43	0.43 ± 0.44	0.47 ± 0.48	0.24 ± 0.21	0.31 ± 0.14	0.30 ± 0.24	0.51 ± 0.41	0.35 ± 0.25	0.38 ± 0.37
*SB-1*	0.01 ± 0.02	0.24 ± 0.29	0.01 ± 0.02	0.00 ± 0.00	0.00 ± 0.00	0.00 ± 0.00	0.00 ± 0.00	0.08 ± 0.18	0.01 ± 0.01
*Sphingobacteriaceae*	0.04 ± 0.06	0.05 ± 0.08	0.05 ± 0.06	0.07 ± 0.08	0.04 ± 0.08	0.11 ± 0.10	0.05 ± 0.07	0.05 ± 0.08	0.08 ± 0.15
*Weeksellaceae*	0.19 ± 0.32	0.13 ± 0.03	0.16 ± 0.16	0.21 ± 0.34	0.07 ± 0.07	0.12 ± 0.11	0.20 ± 0.32	0.09 ± 0.06	0.14 ± 0.13
*Chlamydiae*	0.00 ± 0.00	0.07 ± 0.11	0.04 ± 0.09	0.01 ± 0.02	0.00 ± 0.00	0.06 ± 0.14	0.00 ± 0.00	0.02 ± 0.06	0.05 ± 0.12
*Euryarchaeota*	0.09 ± 0.07	0.03 ± 0.03	0.07 ± 0.08	0.01 ± 0.03	0.09 ± 0.12	0.02 ± 0.02	0.06 ± 0.07	0.07 ± 0.10	0.04 ± 0.06
*Fibrobacteres*	0.05 ± 0.02	0.11 ± 0.17	0.00 ± 0.00	0.01 ± 0.03	0.00 ± 0.00	0.00 ± 0.00	0.01 ± 0.02	0.03 ± 0.10	0.00 ± 0.00
*Fibrobacteraceae*	0.01 ± 0.01	0.06 ± 0.09	0.00 ± 0.00	0.01 ± 0.03	0.00 ± 0.00	0.00 ± 0.00	0.00 ± 0.00	0.02 ± 0.05	0.00 ± 0.00
*TSCOR003-O20*	0.01 ± 0.02	0.05 ± 0.07	0.00 ± 0.00	0.00 ± 0.00	0.00 ± 0.00	0.00 ± 0.00	0.01 ± 0.02	0.01 ± 0.04	0.00 ± 0.00
*Firmicutes*	18.84 ± 4.78	16.98 ± 2.32	21.23 ± 12.06	13.34 ± 6.20	15.22 ± 14.33	14.45 ± 5.87	16.69 ± 5.91	15.81 ± 11.42	17.60 ± 9.71
*Acidaminobacteraceae*	0.03 ± 0.01	0.16 ± 0.25	0.01 ± 0.04	0.00 ± 0.00	0.00 ± 0.00	0.01 ± 0.03	0.01 ± 0.01	0.05 ± 0.14	0.01 ± 0.03
*Aerococcaceae*	0.51 ± 0.49	0.45 ± 0.62	0.37 ± 0.48	0.14 ± 0.20	0.09 ± 0.14	0.08 ± 0.10	0.37 ± 0.43	0.21 ± 0.38	0.21 ± 0.36
*Bacillaceae*	2.00 ± 1.16	2.38 ± 1.55	2.08 ± 1.34	3.20 ± 0.59	3.22 ± 1.23	3.11 ± 1.06	2.47 ± 1.13	2.94 ± 1.31	2.62 ± 1.29
*Clostridiaceae*	0.70 ± 0.61	1.02 ± 1.03	0.81 ± 0.81	0.63 ± 0.60	0.83 ± 1.08	0.39 ± 0.42	0.67 ± 0.59	0.89 ± 1.00	0.58 ± 0.65
*Enterococcaceae*	0.79 ± 1.53	0.15 ± 0.11	0.21 ± 0.22	0.66 ± 0.71	0.39 ± 0.28	0.43 ± 0.15	0.74 ± 1.26	0.31 ± 0.26	0.33 ± 0.21
*Erysipelotrichaceae*	4.83 ± 4.81	3.12 ± 1.57	6.79 ± 10.13	3.22 ± 4.78	4.22 ± 10.11	2.29 ± 3.56	4.20 ± 4.75	3.85 ± 8.05	4.38 ± 7.58
*Lachnospiraceae*	1.00 ± 0.41	1.16 ± 0.67	1.05 ± 0.50	0.59 ± 0.35	0.91 ± 0.82	0.42 ± 0.23	0.84 ± 0.43	1.00 ± 0.74	0.71 ± 0.49
*Lactobacillacea*e	1.36 ± 1.02^a^	0.69 ± 0.12^b^	0.89 ± 0.26^b^	1.12 ± 0.35^a^	0.86 ± 0.28^ab^	0.69 ± 0.20^b^	1.27 ± 1.01^a^	0.80 ± 0.41^ab^	0.79 ± 0.39^b^
*Leuconostocaceae*	0.11 ± 0.12	0.05 ± 0.04	0.08 ± 0.11	0.11 ± 0.05	0.11 ± 0.06	0.17 ± 0.1	0.11 ± 0.10	0.09 ± 0.06	0.13 ± 0.12
*Listeriaceae*	0.00 ± 0.00	0.00 ± 0.00	0.07 ± 0.15	0.00 ± 0.00	0.00 ± 0.00	0.13 ± 0.26	0.00 ± 0.00	0.00 ± 0.00	0.10 ± 0.12
*Mogibacteriaceae*	0.14 ± 0.13	0.15 ± 0.13	0.11 ± 0.14	0.01 ± 0.02	0.03 ± 0.04	0.01 ± 0.03	0.09 ± 0.12	0.07 ± 0.09	0.06 ± 0.10
*Paenibacillaceae*	0.44 ± 0.45	0.37 ± 0.29	0.27 ± 0.30	0.51 ± 0.23	0.77 ± 0.22	0.51 ± 0.27	0.47 ± 0.37	0.63 ± 0.30	0.40 ± 0.31
*Peptostreptococcaceae*	0.11 ± 0.16	0.13 ± 0.07	0.13 ± 0.22	0.08 ± 0.08	0.05 ± 0.05	0.04 ± 0.08	0.10 ± 0.13	0.07 ± 0.06	0.08 ± 0.16
*Planococcaceae*	0.88 ± 0.57	1.59 ± 1.96	0.78 ± 0.29	0.50 ± 0.22	0.64 ± 0.49	0.38 ± 0.24	0.73 ± 0.50	0.96 ± 1.15	0.57 ± 0.33
*Ruminococcaceae*	2.60 ± 2.29	3.02 ± 2.75	1.91 ± 2.40	0.58 ± 0.35	0.48 ± 0.50	0.54 ± 0.39	1.81 ± 2.04	1.33 ± 1.91	1.18 ± 1.77
*Staphylococcaceae*	0.66 ± 0.43	0.79 ± 0.20	0.67 ± 0.62	0.39 ± 0.36	0.28 ± 0.25	0.23 ± 0.17	0.56 ± 0.42	0.45 ± 0.34	0.43 ± 0.48
*Streptococcaceae*	1.68 ± 1.13^b^	0.78 ± 0.21^b^	4.13 ± 1.79^a^	1.02 ± 0.98^b^	1.60 ± 1.68^b^	3.86 ± 1.69^a^	1.43 ± 1.10^ab^	1.33 ± 2.16^b^	3.98 ± 4.21^a^
*Tissierellaceae*	0.53 ± 0.39	0.60 ± 0.49	0.44 ± 0.37	0.26 ± 0.25	0.19 ± 0.16	0.25 ± 0.23	0.42 ± 0.36	0.33 ± 0.34	0.34 ± 0.31
*Turicibacteraceae*	0.26 ± 0.17	0.20 ± 0.19	0.20 ± 0.16	0.17 ± 0.15	0.15 ± 0.12	0.17 ± 0.18	0.22 ± 0.17	0.17 ± 0.14	0.18 ± 0.17
*Fusobacteria*	0.06 ± 0.08	0.00 ± 0.00	0.01 ± 0.01	0.03 ± 0.07	0.07 ± 0.07	0.03 ± 0.04	0.05 ± 0.07	0.05 ± 0.06	0.02 ± 0.03
*Fusobacteriaceae*	0.06 ± 0.08	0.00 ± 0.00	0.00 ± 0.00	0.03 ± 0.07	0.06 ± 0.07	0.03 ± 0.04	0.05 ± 0.01	0.04 ± 0.06	0.02 ± 0.03
*Leptotrichiaceae*	0.00 ± 0.00	0.00 ± 0.00	0.01 ± 0.01	0.00 ± 0.00	0.01 ± 0.01	0.00 ± 0.00	0.00 ± 0.00	0.01 ± 0.02	0.00 ± 0.00
*Proteobacteria*	64.57 ± 7.40	65.16 ± 9.29	66.98 ± 14.25	79.92 ± 6.70	79.57 ± 15.98	80.75 ± 6.43	70.58 ± 10.36	74.77 ± 15.27	74.36 ± 12.67
*Acetobacteraceae*	0.02 ± 0.06	0.00 ± 0.00	0.02 ± 0.06	0.01 ± 0.02	0.04 ± 0.07	0.01 ± 0.02	0.02 ± 0.04	0.03 ± 0.06	0.01 ± 0.05
*Aeromonadaceae*	0.18 ± 0.18	0.36 ± 0.27	0.17 ± 0.16	0.50 ± 0.14	0.32 ± 0.10	0.38 ± 0.16	0.31 ± 0.23	0.33 ± 0.16	0.28 ± 0.19
*Alteromonadaceae*	0.11 ± 0.13	0.36 ± 0.93	0.02 ± 0.09	0.00 ± 0.00	0.00 ± 0.00	0.01 ± 0.04	0.01 ± 0.02	0.19 ± 0.54	0.02 ± 0.04
*Brucellaceae*	0.02 ± 0.03	0.01 ± 0.02	0.01 ± 0.01	0.03 ± 0.05	0.03 ± 0.03	0.03 ± 0.04	0.02 ± 0.04	0.02 ± 0.02	0.02 ± 0.03
*Burkholderiaceae*	0.37 ± 0.71	0.09 ± 0.15	0.11 ± 0.13	0.35 ± 0.48	0.03 ± 0.07	0.23 ± 0.17	0.36 ± 0.62	0.05 ± 0.10	0.17 ± 0.16
*Campylobacteraceae*	0.07 ± 0.13	0.32 ± 0.38	0.02 ± 0.05	0.00 ± 0.00	0.00 ± 0.00	0.01 ± 0.02	0.04 ± 0.10	0.11 ± 0.25	0.01 ± 0.03
*Caulobacteraceae*	0.18 ± 0.23	0.84 ± 1.38	0.23 ± 0.37	0.24 ± 0.27	0.24 ± 0.20	0.18 ± 0.19	0.20 ± 0.25	0.44 ± 0.77	0.20 ± 0.28
*Chromatiaceae*	0.12 ± 0.13	0.06 ± 0.10	0.10 ± 0.10	0.02 ± 0.06	0.02 ± 0.04	0.04 ± 0.12	0.08 ± 0.11	0.03 ± 0.06	0.07 ± 0.11
*Comamonadaceae*	0.76 ± 1.08	0.88 ± 1.17	0.32 ± 0.16	0.29 ± 0.16	0.14 ± 0.10	0.17 ± 0.15	0.57 ± 0.86	0.38 ± 0.70	0.24 ± 0.17
*Desulfovibrionaceae*	0.20 ± 0.24	0.18 ± 0.26	0.17 ± 0.26	0.00 ± 0.00	0.01 ± 0.03	0.01 ± 0.05	0.12 ± 0.21	0.07 ± 0.15	0.09 ± 0.19
*Enterobacteriaceae*	4.98 ± 3.62	4.66 ± 3.80	4.96 ± 3.25	7.73 ± 1.77	7.66 ± 1.16	7.42 ± 1.12	6.06 ± 3.28	6.66 ± 2.58	6.28 ± 2.63
*Halomonadaceae*	0.14 ± 0.20	0.98 ± 1.05	0.13 ± 0.11	0.00 ± 0.00	0.01 ± 0.01	0.02 ± 0.03	0.09 ± 0.17	0.33 ± 0.89	0.07 ± 0.10
*Methylobacteriaceae*	0.37 ± 0.26	0.54 ± 0.56	0.22 ± 0.11	1.20 ± 0.76	0.85 ± 0.60	0.64 ± 0.55	0.69 ± 0.65	0.75 ± 0.57	0.44 ± 0.45
*Moraxellaceae*	22.23 ± 4.24^b^	26.70 ± 8.6^ab^	30.09 ± 4.87^a^	40.49 ± 4.65	39.16 ± 10.86	42.74 ± 3.95	29.37 ± 4.51^b^	35.01 ± 4.09^ab^	36.87 ± 2.15^a^
*Neisseriaceae*	0.13 ± 0.17	0.05 ± 0.08	0.09 ± 0.09	0.12 ± 0.16	0.05 ± 0.08	0.06 ± 0.11	0.12 ± 0.16	0.05 ± 0.08	0.08 ± 0.10
*OM27*	0.00 ± 0.00	0.01 ± 0.02	0.00 ± 0.00	0.00 ± 0.00	0.00 ± 0.00	0.00 ± 0.00	0.00 ± 0.00	0.01 ± 0.01	0.00 ± 0.00
*Oxalobacteraceae*	0.45 ± 0.61	0.24 ± 0.30	0.15 ± 0.21	0.07 ± 0.08	0.08 ± 0.08	0.06 ± 0.07	0.30 ± 0.51	0.13 ± 0.18	0.10 ± 0.16
*Pasteurellaceae*	0.01 ± 0.01^b^	0.09 ± 0.09^b^	3.05 ± 1.79^a^	0.01 ± 0.04^b^	0.00 ± 0.00^b^	1.17 ± 1.08^a^	0.01 ± 0.02^b^	0.03 ± 0.06^b^	2.04 ± 1.30^a^
*Phyllobacteriaceae*	1.03 ± 1.04	0.93 ± 1.43	0.72 ± 0.90	0.15 ± 0.14	0.12 ± 0.12	0.14 ± 0.11	0.69 ± 0.92	0.39 ± 0.83	0.41 ± 0.67
*Pseudomonadaceae*	0.96 ± 1.01	0.38 ± 0.41	0.99 ± 1.31	0.55 ± 0.21	0.49 ± 0.16	0.58 ± 0.21	0.80 ± 0.81	0.45 ± 0.25	0.77 ± 0.91
*Rhodobacteraceae*	0.25 ± 0.23	0.34 ± 0.17	0.24 ± 0.20	0.07 ± 0.09	0.18 ± 0.08	0.09 ± 0.11	0.18 ± 0.20	0.23 ± 0.13	0.16 ± 0.17
*Rhodocyclaceae*	0.39 ± 0.41	0.57 ± 0.45	0.21 ± 0.26	0.02 ± 0.04	0.00 ± 0.00	0.01 ± 0.02	0.24 ± 0.36	0.19 ± 0.36	0.10 ± 0.20
*Sphingomonadaceae*	29.16 ± 5.17^a^	23.28 ± 11.18^ab^	21.38 ± 7.18^b^	25.01 ± 4.24	25.09 ± 4.51	22.86 ± 5.68	27.54 ± 3.48^a^	24.49 ± 4.35^ab^	22.17 ± 2.34^b^
*Succinivibrionaceae*	0.23 ± 0.29	0.16 ± 0.13	0.07 ± 0.10	0.02 ± 0.03	0.02 ± 0.03	0.01 ± 0.03	0.15 ± 0.25	0.06 ± 0.10	0.04 ± 0.07
*Xanthobacteraceae*	2.08 ± 0.58	1.81 ± 0.17	2.95 ± 3.78	2.02 ±0.37	2.07 ± 0.58	2.44 ± 0.48	2.06 ± 0.50	1.99 ± 0.48	2.68 ± 2.55
*Spirochaetes*	0.16 ± 0.19	0.22 ± 0.19	0.14 ± 0.19	0.01 ± 0.01	0.02 ± 0.05	0.02 ± 0.04	0.10 ± 0.16	0.09 ± 0.14	0.08 ± 0.14
*Spirochaetaceae*	0.16 ± 0.19	0.22 ± 0.19	0.14 ± 0.19	0.01 ± 0.01	0.02 ± 0.05	0.02 ± 0.04	0.10 ± 0.16	0.09 ± 0.14	0.08 ± 0.14
*Tenericutes*	0.11 ± 0.24^b^	0.58 ± 0.86^ab^	0.63 ± 0.81^a^	0.00 ± 0.00^b^	0.06 ± 0.13^ab^	0.91 ± 1.06^a^	0.07 ± 0.19^b^	0.23 ± 0.51^ab^	0.78 ± 0.95^a^
*Acholeplasmataceae*	0.06 ± 0.18	0.40 ± 0.70	0.03 ± 0.04	0.00 ± 0.00	0.00 ± 0.00	0.00 ± 0.00	0.04 ± 0.14	0.14 ± 0.40	0.01 ± 0.03
*Anaeroplasmataceae*	0.01 ± 0.01	0.01 ± 0.01	0.00 ± 0.00	0.00 ± 0.00	0.00 ± 0.00	0.00 ± 0.00	0.01 ± 0.01	0.00 ± 0.00	0.00 ± 0.00
*Mycoplasmataceae*	0.04 ± 0.08^b^	0.17 ± 0.17^ab^	0.60 ± 0.80^a^	0.00 ± 0.00^b^	0.06 ± 0.13^ab^	0.91 ± 1.06^a^	0.03 ± 0.07^b^	0.09 ± 0.14^b^	0.77 ± 0.95^a^
*Thermi*	0.27 ± 0.27	0.27 ± 0.26	0.20 ± 0.09	0.18 ± 0.14	0.16 ± 0.15	0.14 ± 0.10	0.23 ± 0.23	0.20 ± 0.19	0.17 ± 0.10
*Deinococcaceae*	0.04 ± 0.04	0.06 ± 0.01	0.05 ± 0.06	0.07 ± 0.07	0.11 ± 0.17	0.05 ± 0.04	0.05 ± 0.05	0.09 ± 0.14	0.05 ± 0.05
*Thermaceae*	0.21 ± 0.24	0.19 ± 0.28	0.14 ± 0.12	0.11 ± 0.15	0.03 ± 0.04	0.08 ± 0.08	0.17 ± 0.21	0.09 ± 0.16	0.11 ± 0.10
*Trueperaceae*	0.01 ± 0.01	0.02 ± 0.03	0.00 ± 0.00	0.00 ± 0.00	0.02 ± 0.05	0.01 ± 0.02	0.01 ± 0.01	0.02 ± 0.04	0.00 ± 0.00
*Verrucomicrobia*	0.47 ± 1.22	0.11 ± 0.05	0.07 ± 0.08	0.73 ± 1.49^a^	0.08 ± 0.09^ab^	0.01 ± 0.04^b^	0.57 ± 1.31	0.09 ± 0.08	0.04 ± 0.07
*Opitutaceae*	0.40 ± 1.19	0.09 ± 0.08	0.01 ± 0.04	0.55 ± 1.24^a^	0.06 ± 0.07^ab^	0.01 ± 0.05^b^	0.46 ± 0.18^a^	0.07 ± 0.07^ab^	0.01 ± 0.03^b^

Different superscripts indicate significant differences within each farm in the same row (*p* < 0.05). HU, healthy udder; MSU, mastitis-suspected udder; SM, subclinical mastitis

**Figure 3.**
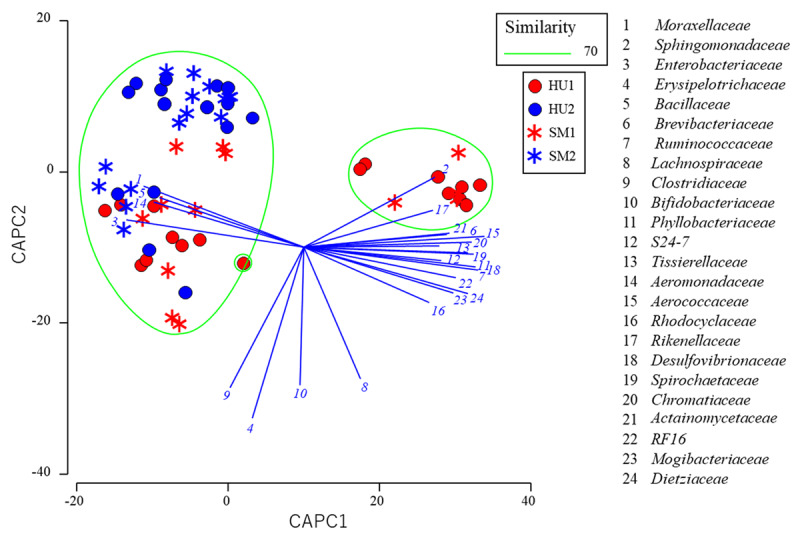
The milk microbiota from two groups of dairy cows with different health statuses of the udder through canonical analysis of principal coordinates (CAPC) plot. The same groups at a 70% similarity level were enclosed in a green circle. Lines denote discriminatory vectors, with a Pearson correlation coefficient > 0.7, and the groupings for the bacterial families.

**Figure 4.**
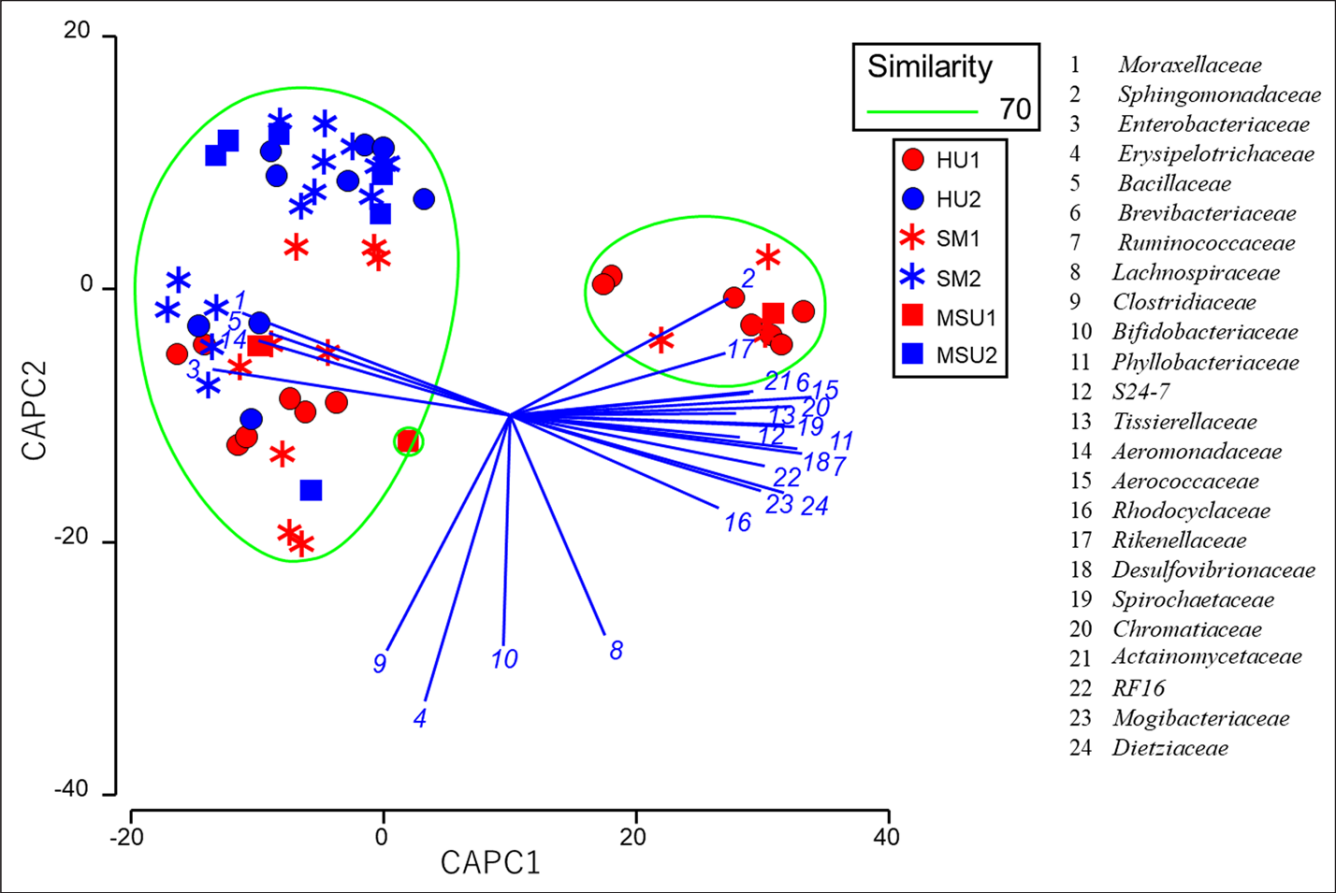
The milk microbiota from three groups of dairy cows with different health statuses of udder through canonical analysis of principal coordinates (CAPC) plot. The same groups at a 70% similarity level were enclosed in a green circle. Lines denote discriminatory vectors, with a Pearson correlation coefficient > 0.7, and the groupings for the bacterial families.

The results from the canonical analysis of principal coordinates (CAPC) with the milk microbiota from two groups of dairy cows with different health statuses indicated that the milk microbiota from healthy dairy cows at farm 1 was grouped with the subclinical mastitis microbiota in two separate areas, whereas the milk microbiota from healthy cows at farm 2 was grouped with the subclinical mastitis microbiota in only one area (Figure 3). Similarly, the findings from 3 groups of cows revealed that the milk microbiota from healthy dairy cows at farm 1 was grouped with the mastitis-suspected udder and/or subclinical mastitis microbiota in two separate areas, while the milk microbiota from healthy cows at farm 2 was grouped with the mastitis-suspected udder and/or subclinical mastitis microbiota in only one area (Figure 4).

## 4. Discussion

In the present study, the average prevalence of milking cows with subclinical mastitis disease of dairy farms in Southern Vietnam was quite high ranging from 43.33–50.00%, consistent with the previous results from dairy cows herd with a range of 30–57% in Ho Chi Minh City, Vietnam [5], but signicicantly better than that of older findings with the subclinical mastitis at 63.2–88.6% in Southern Vietnam [9]. It appears that subclinical mastitis prevalence is lower in dairy cows in Southern Vietnam; however, mastitis significantly reduces milk yield (>15%) and quality parameters, thereby affecting productivity and profitability in the dairy industry [2, 3, 4, 5, 6, 7, 8]. In addition, there are significant negative effects of milk from dairy cows with mastitis on consumer health [12, 13, 14], due to bacteria and their toxins, or to antibiotic residues in milk following antibiotic treatment [15, 16]. Therefore, it is important to determine which bacteria are the main pathogens (significant reduction or increase between healthy and subclinical mastitis cows) through milk microbiota for the current subclinical mastitis of dairy farms under Vietnam’s weather conditions to contribute to preventing mastitis and improving milk productivity/quality for the sustainable development of the dairy industry in Vietnam.

Our results from Illumina MiSeq sequencing indicated that there were significant unchanges of the four major phyla (*Proteobacteria, Firmicutes, Actinobacteria*, and *Bacteroidetes*) between two groups (healthy udder *vs*. subclinical mastitis) or among three groups of cows (healthy udder, mastitis-suspected udder vs subclinical mastitis), which was contrasted to the findings from recent publications [33, 34]. As described above, however, more than 135 bacterial species and 20 common pathogens have been detected in cows with mastitis [35, 36]. Interestingly, the current findings in this investigation indicated that abundances of *Tenericutes* were significantly increased and those of *Verrucomicrobia* were remarkably decreased in milk from mastitis-suspected udders and/or subclinical mastitis, as compared to those in healthy udder groups. Therefore, it seems that the *Tenericutes* phylum is also the main pathogen causing subclinical mastitis in dairy cows in Southern Vietnam, in addition to previously discovered main pathogenic phyla such as *Proteobacteria, Firmicutes, Actinobacteria*, and *Bacteroidetes*. It also suggests that the Verrucomicrobia phylum may not remain at a stable, balanced level when the abundance of pathogenic phyla increases significantly.

Furthermore, our current results indicate that *Moraxellaceae, Sphingomonadaceae, Enterobacteriaceae, Erysipelotrichaceae*, and *Streptococcaceae* were the five most common families in milk from dairy cows, regardless of udder health status, consistent with previous findings [37]. Recently, it has been demonstrated that *Staphylococcaceae* (*Staphylococcus* spp., *Staphylococcus aureus*), *Enterobacteriaceae* (*Escherichia coli, Klebsiella* spp.), and *Streptococcaceae* (*Streptococcus* spp.) are the main agents for subclinical and clinical mastitis, affecting milk quality of dairy cows [20, 23].

Therefore, not only the previous isolated families (such as *Streptococcaceae, Staphylococcaceae, Enterobacteriaceae*) but also the other agents of *Mycobacteriaceae, Moraxellaceae, Pasteurellaceae*, and *Mycoplasmataceae* seem to be the main causes for subclinical mastitis, since the quantity of them (*Mycobacteriaceae, Streptococcaceae, Moraxellaceae, Pasteurellaceae*, and *Mycoplasmataceae*) in milk significantly increased from healthy udder to subclinical mastitis cows. *Streptococcaceae* are well known as a common cause of subclinical mastitis in cows, driven by environmental factors and management practices [38, 39, 40, 41]. It has also been reported that *Mycobacteriaceae*, a common cause of mastitis from living environments entering the mammary gland, can significantly increase in milk from subclinical mastitis cows [42]. *Mycoplasmataceae* are well known to increase in milk during subclinical mastitis in dairy cows [8], significantly impact milk yield and quality parameters, and consequently lead to economic losses for the dairy industry [43, 44]. In addition, it has been demonstrated that, although Pasteurellaceae are often considered less common mastitis-causing pathogens, they remain a common potential cause of subclinical mastitis [45, 46]. While *Moraxellaceae* are not typically the primary agents of mastitis in dairy cows, they can be associated with subclinical mastitis [47], probably due to disrupted rumen microbiome, development of mammary gland inflammation, and/or a disrupted immune response triggered by other pathogens and/or stressors [48]. Since the increase in these pathogenic bacteria in milk results in a significant reduction in the relative abundance of beneficial bacteria [33], the families *Rikenellaceae, Lactobacillaceae, Sphingomonadaceae*, and *Opitutaceae* in the current study significantly decreased in subclinical mastitis cows.

In contrast, our current findings revealed no significant differences in milk microbiota among families of healthy and mastitis-suspected cows, although there were clear changes in the phyla *Tenericutes* and *Verrucomicrobia*. Therefore, the levels of SCC in milk from 200,000 to 400.000 cells/ml (cows with mastitis-suspected udder) seem not to be enough to cause compositional differences of milk microbiota as compared to the milk microbiota from dairy cows tested with SCC in milk < 200,000 cells/ml (cows with healthy udder).

Certain limitations of this study should be acknowledged. First, the sample size (60 milk samples from 60 cows) and the number of farms surveyed (2 farms) were relatively restricted, primarily due to the substantial costs associated with high-throughput next-generation sequencing technologies, which constrained the ability to expand sampling across a broader geographical range in this initial investigation. Consequently, while the identified core microbiome is robust for the sampled population, caution should be applied when generalizing these findings to the entire dairy cattle population in Vietnam. Second, milk samples were collected as composite samples from all four quarters to represent the cow-level health status. We recognize that this pooling method implies a dilution effect, which might mask specific pathogen signals from a single infected quarter or lead to an underestimation of localized intramammary infections compared to quarter-level sampling. Third, although current bioinformatics pipelines favor Amplicon Sequence Variants (ASVs) for higher resolution, the 97% OTU clustering (QIIME 1.9.0) employed here remains a valid approach for establishing broad taxonomic profiles. Given the exploratory nature of this investigation, which serves as the first comprehensive profile of milk microbiota in this specific region, this method provides a robust taxonomic overview sufficient to identify major compositional shifts and core bacterial communities associated with udder health status.

## 5. Conclusions

This study confirms a high prevalence of subclinical mastitis in dairy herds in Southern Vietnam. While the core milk microbiome is dominated by the phyla *Proteobacteria, Firmicutes, Actinobacteria*, and *Bacteroidetes*, as well as taxa in *Moraxellaceae, Sphingomonadaceae, Enterobacteriaceae, Erysipelotrichaceae*, and *Streptococcaceae*, the disease state is characterized by a complex dysbiosis. We identified that subclinical mastitis is driven not only by traditional pathogens (*Streptococcaceae, Staphylococcaceae, Enterobacteriaceae*) but also by the enrichment of other potential taxa, including *Mycobacteriaceae, Moraxellaceae, Pasteurellaceae*, and *Mycoplasmataceae*. Notably, the milk microbiota of mastitis-suspected cows (intermediate SCC) remained statistically similar to that of healthy udders, suggesting that moderate SCC elevations do not necessarily reflect a disruption in the microbial community. Based on these findings, we recommend that dairy farmers in tropical regions such as Vietnam should not rely solely on visual inspection or high SCC thresholds. Regular screening for mastitis-suspected cows (SCC 200,000–400,000 cells/ml) and implementing stricter hygiene protocols during milking are crucial to prevent progression to subclinical and clinical mastitis. Future farm management strategies should consider integrating microbiome monitoring as an early warning tool for udder health.

## Data Availability

The data presented in this study are available from the corresponding author upon reasonable request.

## List of abbreviations

ANOVA, analysis of variance; CAPC, canonical analysis of principal coordinates; HU, healthy udders; MSU, mastitis-suspected; SCC, somatic cell counts; SM, subclinical mastitis; *16S rRNA*, 16S ribosomal ribonucleic acid; ASV, Amplicon sequence variant; bp, base pairs; CFU, Colony forming unit; DNA, Deoxyribonucleic acid; g, relative centrifugal force; h, hour; min, minute; ml, milliliter; mM, millimolar; NGS, next-generation sequencing; NM, non-mastitis; OTU, Operational taxonomic unit; PBS, Phosphate buffered saline; PCR, Polymerase chain reaction; rpm, revolutions per minute; V3-V4, variable regions 3 and 4 of the *16S rRNA* gene; μl, microliter; °C, degrees Celsius.
